# Validation of a Novel, Low‐Cost, Portable MRI‐Compatible Exercise Device in Healthy Volunteers and Patients With Pulmonary Hypertension

**DOI:** 10.1002/nbm.70412

**Published:** 2026-10-04

**Authors:** Ruta Virsinskaite, James T. Brown, Tushar Kotecha, Darren Bower, Jennifer A. Steeden, Javier Montalt‐Tordera, Olivier Jaubert, Marianna Fontana, J. Gerry Coghlan, Daniel S. Knight, Vivek Muthurangu

**Affiliations:** ^1^ National Pulmonary Hypertension Service Royal Free London NHS Foundation Trust London UK; ^2^ Department of Cardiac MRI Royal Free London NHS Foundation Trust London UK; ^3^ Institute of Cardiovascular Science University College London London UK; ^4^ Engineering Department Royal Free London NHS Foundation Trust London UK; ^5^ Division of Medicine University College London London UK

**Keywords:** exercise CMR device, pulmonary hypertension, systemic sclerosis

## Abstract

The value of exercise cardiovascular magnetic resonance (CMR) has been shown in many clinical scenarios. We have developed a novel MR‐compatible exercise device and aim to validate it against the reference standard MR‐conventional ergometer. The novel device consisted of two half‐pipes fixed to a wooden base, with participants wearing knee‐length socks with a 0.5 kg weight in each sock. Increased workload was achieved by increasing the rate of alternating leg flexion and extension in time with a bleep sound of increasing frequency. Twenty subjects (10 healthy volunteers, 10 patients with pulmonary arterial hypertension) performed two CMR‐augmented cardiopulmonary exercise tests (CMR/CPET) using the novel exercise device and a conventional ergometer in a randomised order. Comparing peak metrics elicited on both exercise devices, there was a moderate correlation in peak oxygen consumption (VO_2_, *r* = 0.86, *p* < 0.001), cardiac output (CO, *r* = 0.73, *p* < 0.001), stroke volume (SV, *r* = 0.90, *p* < 0.001), peak heart rate (HR, *r* = 0.65, *p* = 0.002) and peak arteriovenous oxygen content gradient (ΔavO_2_, *r* = 0.71, *p* < 0.001). However, all metrics were significantly lower with the novel device. Both devices were able to elicit statistically significant differences in VO_2_, HR and right ventricular ejection fraction (RVEF) between patients and healthy subjects (*p* ≤ 0.04). We have created a simple, easy to use and affordable exercise device for the CMR environment. This may encourage greater dissemination of exercise CMR in clinical and research practice.

## Graphical abstract

We developed a simple, low‐cost MR‐compatible exercise device as an alternative to commercial ergometers. In this proof‐of‐concept study, it evoked a robust stress response during MR‐augmented cardiopulmonary exercise testing and distinguished pulmonary arterial hypertension patients from healthy controls. Its portability and low build cost may encourage wider dissemination of exercise CMR.

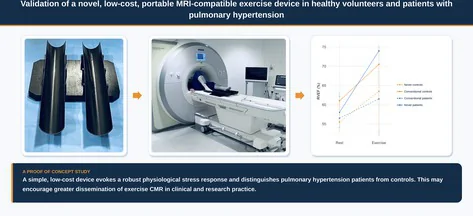

## 1 Background

Exercise cardiovascular magnetic resonance (CMR) is increasingly used to evaluate cardiovascular reserve. Several studies have shown that exercise CMR can be used to unmask underlying cardiac dysfunction, as well as to investigate the causes of exercise intolerance [1, 2, 3, 4]. This is particularly true in pulmonary arterial hypertension (PAH) where exercise CMR has been used to assess contractile reserve [5, 6] and has been shown to be predictive of mortality [7]. However, exercise CMR has been slow to enter routine clinical practice due to three main factors: (i) excessive motion/breathing during exercise imaging, (ii) the cost of MR‐compatible exercise equipment and (iii) the complexity of set‐up and performing exercise CMR protocols.

Advances in real‐time and accelerated CMR have made imaging during exercise progressively easier, which should aid clinical translation. However, broader adoption of exercise CMR is still limited by the cost (~$40K) and the cumbersome nature of commercial MR‐compatible ergometers. Several studies have successfully demonstrated ‘in‐house’ ergometers [8, 9, 10], but most are complex and would be difficult to manufacture at other sites. In addition, they are not necessarily easier to use than commercial ergometers, which is a significant limitation. Therefore, we have developed a portable, low‐cost and easy‐to‐use MR‐compatible exercise device that can be easily reproduced/manufactured in any institution. The aim of this proof‐of‐concept study was to compare our novel device against a commercial MR‐conditional ergometer in both healthy subjects and PAH patients, using a previously published combined CMR and cardiopulmonary exercise testing (CPET) protocol [5, 11]. This was undertaken to evaluate whether a simple, low‐cost, MR‐compatible alternative could provide comparable physiological stress within the constraints of the MR environment.

## 2 Methods

### 2.1 Study Population

Ten asymptomatic healthy volunteers with no previous medical history of cardiovascular or lung pathology (as assessed by careful questioning of medical history) and ten patients with PAH were recruited. Patients were recruited from the Royal Free Hospital National Pulmonary Hypertension Service. Diagnoses were as follows: connective tissue disease associated PAH = 7, portopulmonary hypertension = 2, idiopathic PAH = 1. Inclusion criteria were (i) clinical diagnosis of pulmonary hypertension confirmed by right‐heart catheterisation for patient groups and (ii) age 18–70 years. Exclusion criteria were (i) general contraindications to CMR scanning, (ii) contraindications to performing exercise test (unstable symptoms, including angina, exertional syncope, WHO class IV symptoms and musculoskeletal disease preventing exercise) and (iii) changes in targeted PAH therapy within 3 months or during the experimental period. The study was approved by UK National Health Service, Health Research Authority, Research Ethics Committee and the study conformed to the declaration of Helsinki (IRAS project ID 226101; REC reference 17/LO/1499, National Health Service Health Research Authority UK CRN 058274). All subjects provided written informed consent.

We compared exercise CMR using our new device to exercise CMR performed on a conventional MR‐compatible supine bike ergometer in both healthy controls and PAH patients. The two devices were tested on separate days in a randomised order with a minimum time between tests of 1 week and maximum of 6 weeks.

Full resuscitation facilities were available during testing and each subject's vectorcardiogram (VCG) and oxygen saturation were monitored continuously using the in‐built system in the CMR scanner (allowing assessment of rate and rhythm but not identification of ischaemia). All patients had peripheral venous access during testing for use in resuscitation protocols in the event of clinical instability.

### 2.2 Novel Exercise Device

The novel MR‐compatible exercise device (Figure 1A) consisted of two 83.3‐cm‐long and 11.2‐cm‐wide half‐pipes (made from commercially available plastic roof guttering) fixed to a wooden base (using wooden brackets and adhesive without any ferromagnetic components). Photographs of the device including dimensions are shown in Figure S1. Our device was designed to fit into the recess of the scanner table, which is usually occupied by a cushion (Figures 1B and S1). However, heavy‐duty Velcro can also be used to secure the device to the table in a stable manner as demonstrated in Figure S2. Subjects wore knee‐length thromboembolic deterrent (TED) stockings with a 0.5‐kg sandbag placed in the front of each sock. The TED stockings provided low friction movement due to the smooth nature of the heel material and were able hold the weight in place due to the elasticity. The 0.5‐kg weight was chosen empirically after preliminary testing in healthy volunteers. Exercise was achieved by alternating leg flexion and extension in time with a bleep sound that increased in frequency every minute from 60 bpm to a plateau of 180 bpm (ramped protocol in Figure S3). Importantly, this exercise could be performed while the subject was in the scanner bore allowing imaging during active exercise. A video demonstrating the exercise technique is included in Video S1, as is the 18‐min sound file containing the ramped bleep sounds (Audio S1).

**FIGURE 1:**
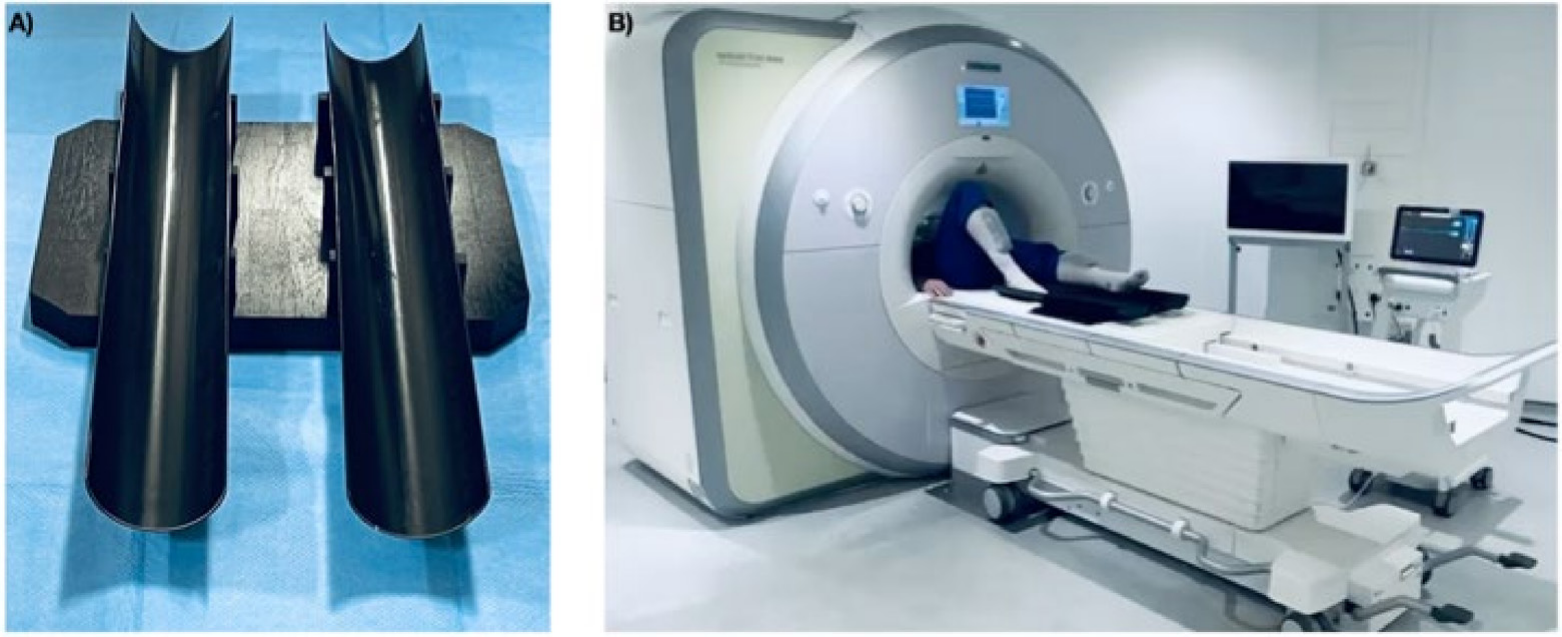
(A) The novel exercise device. (B) Subject exercising using the novel exercise device.

### 2.3 Conventional Ergometer

We compared our novel device with a commercially available MR‐compatible supine rotary cycle ergometer (Lode BV, Groningen, the Netherlands). Exercise was achieved by rotary pedalling at approximately 60–70 rpm with a ramped protocol of increasing resistance as previously described [5]. This device also allowed imaging during active exercise with the patient in the scanner bore. It should be noted that the legs were in an elevated position (strapped into pedals) throughout the scan.

### 2.4 Exercise Protocol

Exercise on both devices was performed until exhaustion (peak exercise), defined as an inability to maintain cadence or a verbal indication from the subject that they were unable to continue. During exercise continuous breath‐by‐breath gas exchange analysis (CPET) was performed and real‐time CMR aortic flow and biventricular volumetric data were acquired at rest and peak exercise.

Breath‐by‐breath gas exchange analysis was performed using a commercial CPET system (Ultima, MedGraphics, St Paul, Minnesota, USA). The analyser was placed in the CMR control room and attached to a facemask (Hans Rudolph, Kansas City, Kansas, USA) via a set of modified nonferromagnetic CMR‐compatible sampling tubes (umbilicus). The umbilicus had a length of 10 m, allowing it to reach the patient from the control room through the waveguide. Gas and flow calibrations were performed before each test and at least 30 min after system initiation. All measurements were taken at body temperature and ambient pressure.

Real‐time CMR was performed on a standard (length 1200 mm, width 489 mm and height 63 mm) 1.5‐T CMR scanner (Magnetom Aera, Siemens Healthineers, Erlangen, Germany) using two six‐element coils (one spinal matrix, one body matrix). Aortic flow was measured using a variable density golden‐angle spiral real‐time phase‐contrast MR (PCMR) sequence with low latency deep learning (DL) reconstruction and segmentation (parameters: slice thickness = 6 mm, velocity encoding = 300 cm/s, temporal resolution = 35 ms, spatial resolution = 2.1 × 2.1 mm, acquisition time = 10 s) [12]. Assessment of left ventricular (LV) and right ventricular (RV) volumes was performed using 2D multislice sorted golden‐angle radial real‐time balanced steady‐state free precession (bSSFP) sequence with DL reconstruction (parameters: slice thickness = 8 mm, temporal resolution ~31 ms, spatial resolution = 1.7 × 1.7 mm, no. of slices = 12, acquisition time = 24 R‐R intervals) [13].

### 2.5 Data Analysis

All post‐processing of the reconstructed images was performed using ‘in‐house’ plug‐ins for the open‐source OsiriX DICOM software version 9.0.1 (OsiriX Foundation, Geneva, Switzerland) [14]. PCMR flow data were automatically segmented as part of reconstruction with manual operator correction if required [12]. Stroke volume (SV) was calculated by integrating the flow curve across a single R‐R interval. Heart rate (HR) was derived from the real‐time flow imaging analysis, which allowed beat‐to‐beat assessment during rest and peak exercise. Cardiac output (CO) was given by SV × HR. Biventricular endocardial borders were traced manually, excluding papillary muscle and trabeculae from the blood pool, on the short‐axis images at end‐diastole and end‐systole, identified by visual assessment of the largest and smallest cavity areas, respectively. LV and RV end‐diastolic volume (EDV) and end‐systolic volume (ESV) and ejection fraction (EF) were determined as (EDV − ESV)/EDV × 100. All measurements were reported by an experienced clinical CMR specialist blinded to the clinical information. The VO_2_ and respiratory exchange ratio (RER) measurements were time‐registered to CMR data. Arteriovenous oxygen content gradient was calculated as ΔavO_2_ = VO_2_/CO.

### 2.6 Statistical Analysis

All statistical analyses were performed using R (version 4.1.2, The R Foundation for Statistical Computing, Vienna, Austria). Data were examined for normality using the Shapiro–Wilk normality test. Descriptive statistics were expressed as mean ±standard deviation for normally distributed data and median (interquartile range) for non‐normally distributed data. Agreement between metrics measured while undergoing exams on both exercise devices was assessed using Spearman rank correlation coefficient and Bland–Altman analysis, with the significance of the bias assessed using a paired *t*‐test. Differences in CMR/CPET metrics between rest and exercise (for both devices) were assessed using paired *t*‐tests (normal data) and Wilcoxon signed rank tests (non‐normal data), while differences between patients and healthy controls were evaluated using *t*‐tests (normal data) and Mann–Whitney tests (non‐normal data). Sex distribution between the groups was assessed using the chi‐squared test. A *p* value < 0.05 was considered statistically significant.

## 3 Results

### 3.1 Demographics and Exercise Feasibility

Compared with healthy volunteers, patients were older (48 ± 14 vs. 29 ± 9 years, *p* = 0.004) and predominantly female (70% vs. 40%, *p* = 0.04). The average height for all participants was 1.69 ± 0.09 m (maximum 1.84 m, minimum 1.50 m) with average body mass index (BMI) of 24.76 ± 3.8 kg/m^2^. All subjects successfully completed both exercise protocols until exhaustion, and no subjects required medical intervention. All subjects (irrespective of height or BMI) were able to perform exercise on the novel device without their legs being obstructed by the scanner bore. Peak RER ≥ 1.0 was achieved in all participants while exercising on the novel exercise apparatus. However, three patients who exercised to exhaustion on the conventional ergometer did not achieve anaerobic threshold (RER < 1.0). The exercise time was significantly shorter with the novel exercise device for both healthy controls and patients (healthy control: conventional 15.02 ± 4.15 min, novel 10.27 ± 4.75 min, *p* < 0.001; patients: conventional 8.35 ± 2.42 min, novel 6.65 ± 3.48 min, *p* = 0.002).

### 3.2 Resting CMR

Comparison of resting volumes on both devices (Table 1) demonstrated good agreement between LV volumes (LVEDV, *r* = 0.94, *p* < 0.001; LVESV, *r* = 0.88, *p* < 0.001), RV volumes (RVEDV, *r* = 0.93, *p* < 0.001; RVESV, *r* = 0.87, *p* < 0.001) and SV (*r* = 0.76, *p* < 0.001). However, resting SV was 9.5% (*p* = 0.01) lower on the novel device, and there was only moderate correlation for CO (*r* = 0.54, *p* = 0.02), LVEF (*r* = 0.54, *p* = 0.01) and RVEF (*r* = 0.56, *p* = 0.01).

**TABLE 1:** Resting CMR metrics when comparing conventional ergometer and novel device.

Variable	*r*	*p*	Bias	LOA	*p*
VO_2_	0.47	0.04	−0.0052	−0.14 to 0.13	0.73
HR	0.76	< 0.001	1.30	−20.82 to 23.42	0.61
SV	0.76	< 0.001	−8.20	−33.96 to 17.56	0.01
CO	0.54	0.02	−0.53	−3.40 to 2.34	0.12
ΔavO_2_	−0.07	0.77	0.47	−2.78 to 3.73	0.22
LVEF	0.54	0.01	−0.55	−11.20 to 10.10	0.66
LVEDV	0.94	< 0.001	0.20	−16.96 to 17.36	0.92
LVESV	0.88	< 0.001	1.35	−13.04 to 15.74	0.42
RVEF	0.56	0.01	−0.35	−11.17 to 10.47	0.78
RVEDV	0.93	< 0.001	−2.05	−29.47 to 25.37	0.52
RVESV	0.87	< 0.001	0.30	−22.06 to 22.66	0.91

Abbreviations: ΔavO_2_—tissue oxygen extraction, CO—cardiac output, HR—heart rate, LOA—limits of agreement, LVEDV—left ventricular end‐diastolic volume, LVEF—left ventricular ejection fraction, LVESV—left ventricular end‐systolic volume, *p*—significant differences between novel and conventional devices, RVEDV—right ventricular end‐diastolic volume, RVEF—right ventricular ejection fraction, RVESV—right ventricular end‐systolic volume, SV—stroke volume, VO_2_—oxygen consumption.

### 3.3 Exercise CMR/CPET

When comparing peak exercise CMR/CPET metrics obtained using both exercise devices (Table 2), there was a good/moderate correlation in peak VO_2_ (*r* = 0.86, *p* < 0.001, Figure 2A), peak CO (*r* = 0.73, *p* < 0.001, Figure 2B), peak SV (*r* = 0.90, *p* < 0.001, Figure 2C), peak HR (*r* = 0.65, *p* = 0.002) and peak ΔavO_2_ (*r* = 0.71, *p* < 0.001). However, all these metrics were significantly lower (5%–37%, *p* ≤ 0.04) on the novel device, with relatively wide limits of agreement (Table 2, Figure 2D–F). There was also good/moderate correlation in peak RV and LV volumes (*r* ≥ 0.71, *p* < 0.001). Although there was no significant bias in RV volumes between the two devices (Table 2), peak LV volumes were significantly higher using the novel device (between 6% and 18%, *p* ≤ 0.04). There was a trend towards significant correlations in peak RVEF and LVEF (RVEF: *r* = 0.42, *p* = 0.06, Figure 3A and LVEF: *r* = 0.39, *p* = 0.09, Figure 3B), with no significant bias (Figure 3C,D).

**TABLE 2:** Peak CMR metrics when comparing novel device and conventional ergometer.

Variable	*r*	*p*	Bias	LOA	*p*
VO_2_	0.86	< 0.001	−0.42	−1.35 to 0.51	< 0.001
HR	0.65	0.002	−8.85	−44.89 to 27.19	0.04
SV	0.90	< 0.001	−4.40	−22.31 to 13.51	0.04
CO	0.73	< 0.001	−1.29	−5.86 to 3.28	0.02
ΔavO_2_	0.71	< 0.001	−2.53	−7.72 to 2.66	< 0.001
LVEF	0.39	0.09	−2.80	−15.07 to 9.47	0.06
LVEDV	0.85	< 0.001	8.15	−24.99 to 41.29	0.04
LVESV	0.71	< 0.001	6.50	−17.14 to 30.14	0.03
RVEF	0.42	0.06	0.30	−13.95 to 14.55	0.86
RVEDV	0.91	< 0.001	4.80	−23.98 to 33.58	0.16
RVESV	0.79	< 0.001	1.15	−26.39 to 28.69	0.72

Abbreviations: ΔavO_2_—tissue oxygen extraction, CO—cardiac output, HR—heart rate, LOA—limits of agreement, LVEDV—left ventricular end‐diastolic volume, LVEF—left ventricular ejection fraction, LVESV—left ventricular end‐systolic volume, *p*—significant differences between novel device and conventional ergometer, RVEDV—right ventricular end‐diastolic volume, RVEF—right ventricular ejection fraction, RVESV—right ventricular end‐systolic volume, SV—stroke volume, VO_2_—oxygen consumption.

**FIGURE 2:**
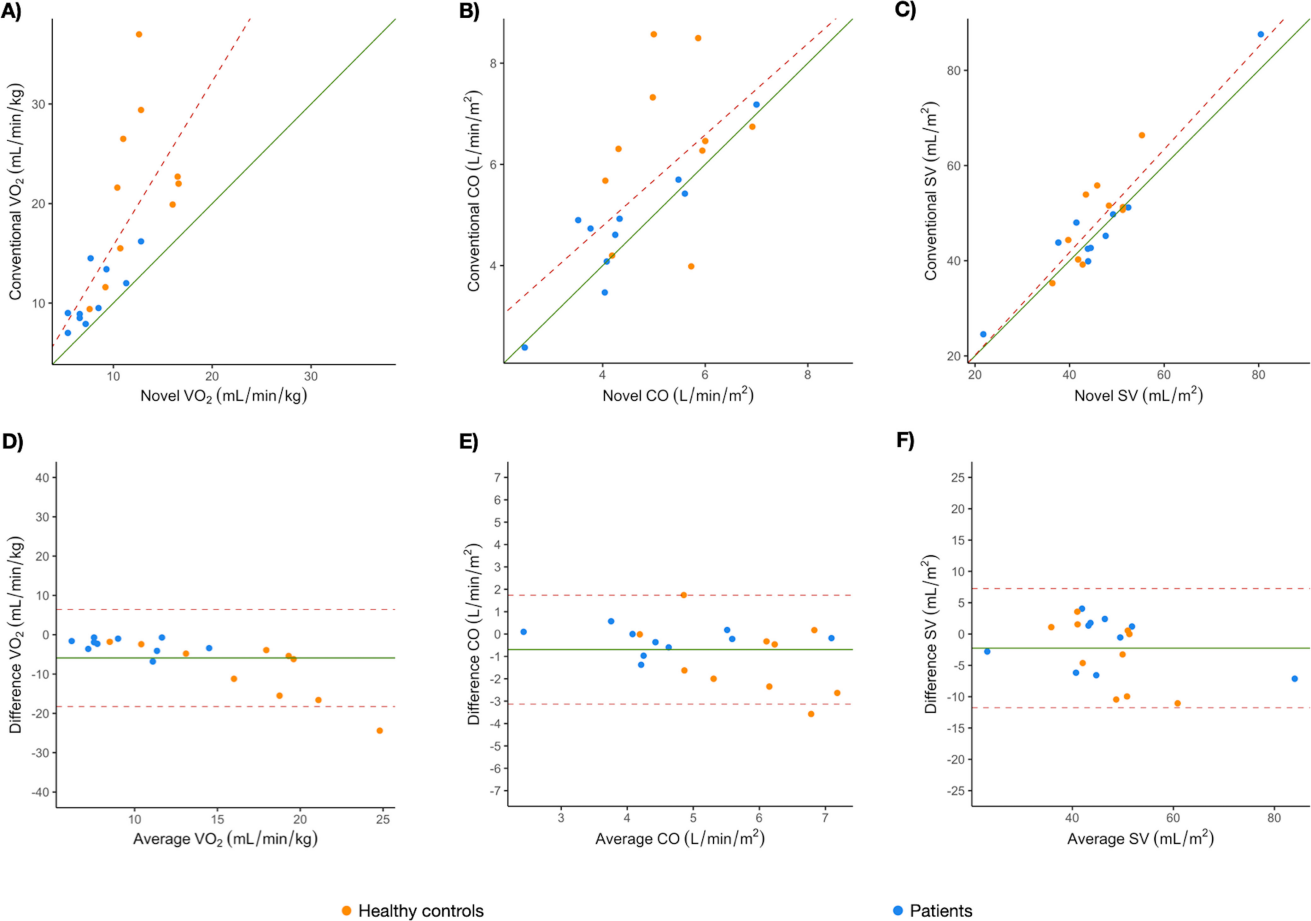
(A–C) Scatter plots of peak haemodynamic parameters acquired using the novel device and conventional MR ergometer. (A) Oxygen consumption (VO_2_). (B) Cardiac output (CO). (C) Stroke volume (SV). (D–F) Bland–Altman charts comparing haemodynamic parameters acquired using the novel device and conventional MR ergometer. (D) VO_2_. (E) CO. (F) SV.

**FIGURE 3:**
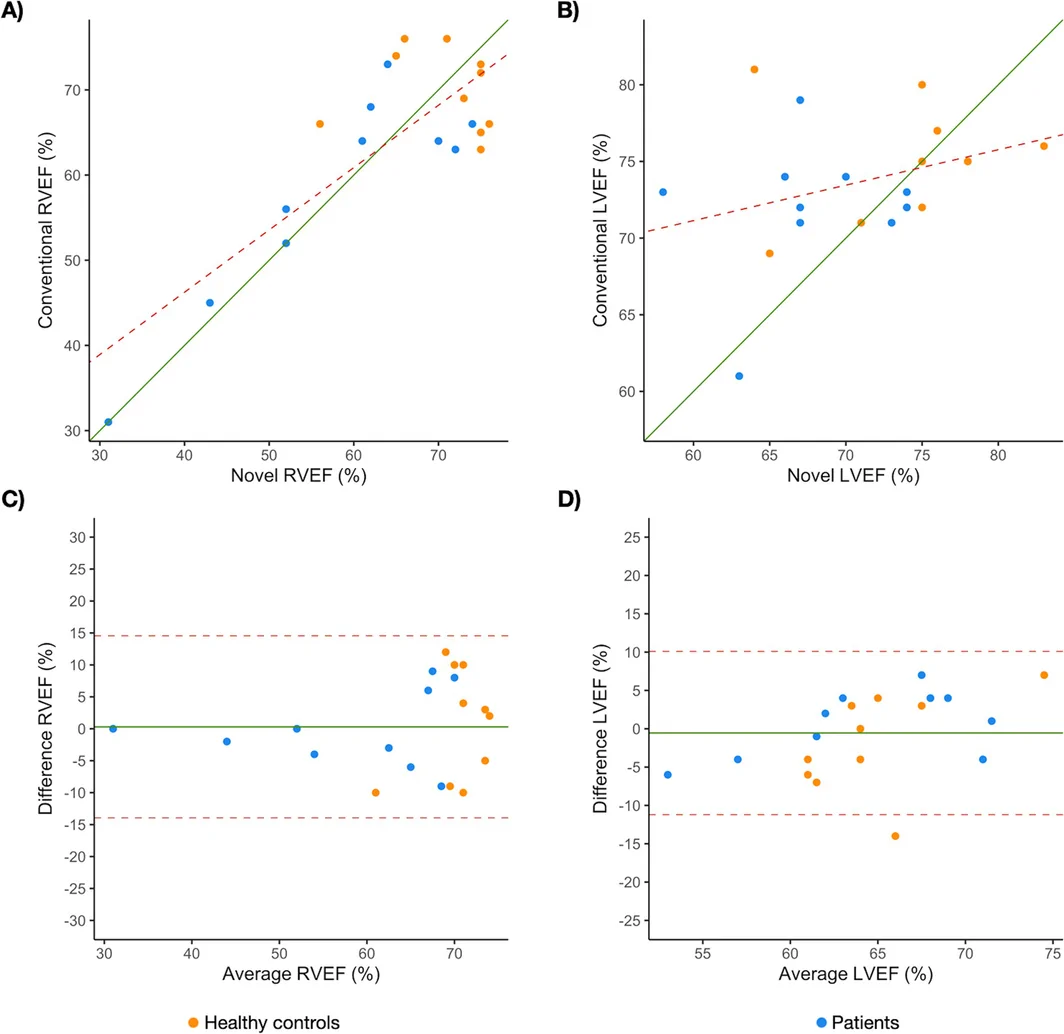
(A,B) Scatter plots of haemodynamic parameters acquired using the novel device and conventional MR ergometer. (A) Right ventricular ejection fraction (RVEF). (B) Left ventricular ejection fraction (LVEF). (C,D) Bland–Altman charts comparing haemodynamic parameters acquired using the novel device and conventional MR ergometer. (C) RVEF. (D) LVEF.

### 3.4 Distinguishing Patients From Controls With Both Protocols

Peak exercise metrics for healthy controls and patients using both exercise devices are shown in Table 3. During exercise VO_2_ and HR significantly increased in both patients and healthy controls on both devices (*p* < 0.001). In addition, patients had significantly lower peak VO_2_ and peak HR than healthy volunteers while exercising on both devices (VO_2_—conventional *p* < 0.001; novel *p* = 0.003, Figure 4A; HR—conventional *p* = 0.001; novel *p* = 0.04).

**TABLE 3:** Exercise CMR metrics for novel device and conventional ergometer.

Variable	PT conventional	HC conventional	PT novel	HC novel
VO_2_	0.6 (0.5–0.9)	1.5 (1.1–1.9)	0.5 (0.4–0.7)	0.8 (0.8–1.0)
HR	101.4 ± 15.2	130.7 ± 19.1	98.1 ± 15.2	116.3 ± 15.9
SV	76.5 (69.8–89.5)	94.0 (69.8–107.0)	77.5 (69.0–85.8)	85.0 (73.8–93.5)
CO	8.4 ± 2.7	11.9 ± 3.2	7.8 ± 2.5	9.9 ± 2.2
ΔavO_2_	8.7 ± 1.9	12.9 ± 2.5	7.2 ± 2.7	9.3 ± 2.5
LVEF	72.0 ± 4.5	75.3 ± 3.8	67.9 ± 5.1	73.8 ± 5.8
LVEDV	122.0 (98.5–136.0)	111.0 (104.0–145.0)	128.0 (107.0–150.0)	134.0 (112.0–156.0)
LVESV	29.5 (27.2–36.8)	26.5 (23.5–33.5)	36.5 (32.2–49.2)	32.5 (27.2–45.5)
RVEF	63.5 (53.0–65.5)	70.5 (66.0–73.8)	61.5 (52.0–68.5)	74.0 (67.2–75.0)
RVEDV	142.0 (132.0–191.0)	118.0 (108.0–155.0)	145.0 (138.0–197.0)	129.0 (110.0–164.0)
RVESV	55.0 (45.5–96.5)	37.0 (34.0–47.0)	60.5 (49.0–73.0)	33.5 (28.0–56.2)

Abbreviations: ΔavO_2_ —tissue oxygen extraction, CO—cardiac output, HC—healthy controls, HR—heart rate, LVEDV—left ventricular end‐diastolic volume, LVEF—left ventricular ejection fraction, LVESV—left ventricular end‐systolic volume, PT—patients, RVEDV—right ventricular end‐diastolic volume, RVEF—right ventricular ejection fraction, RVESV—right ventricular end‐systolic volume, SV—stroke volume, VO_2_—oxygen consumption.

**FIGURE 4:**
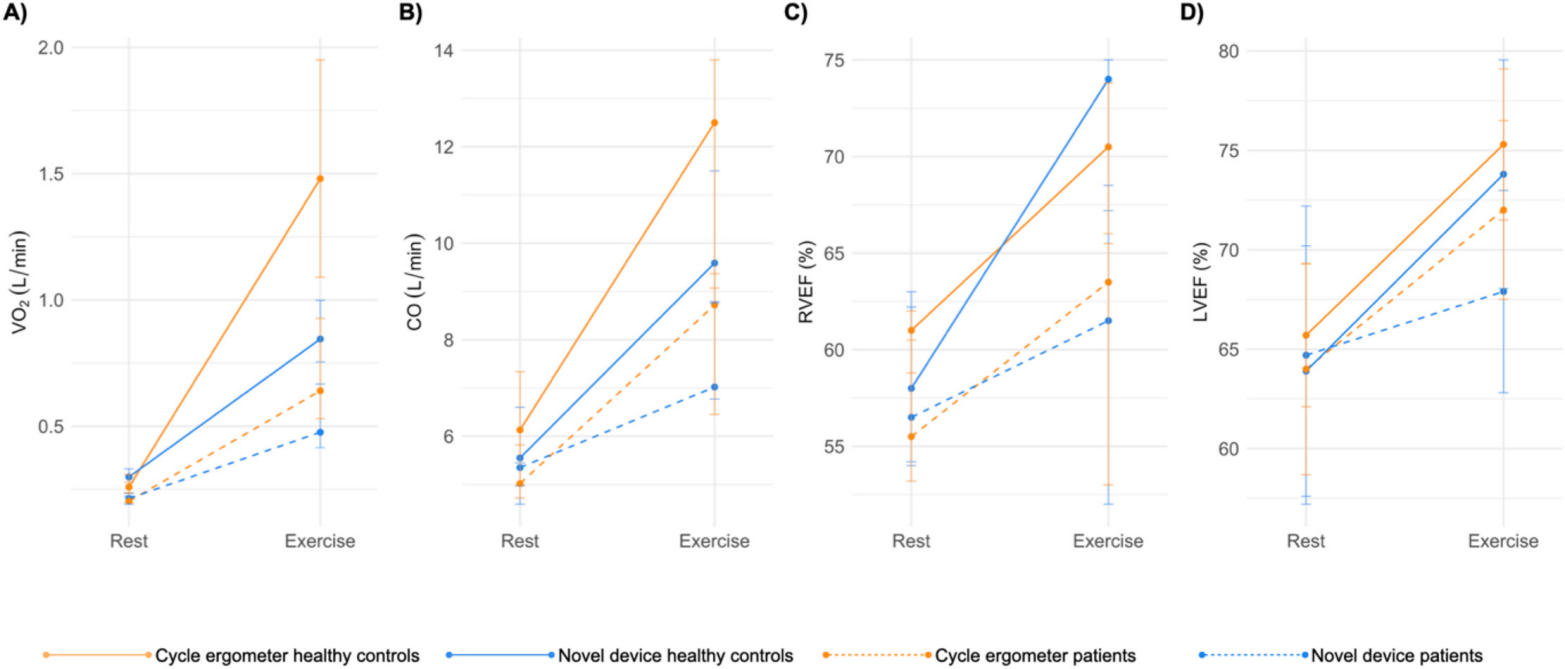
CMR/CPET metrics at rest and peak exercise on both devices for patients and healthy controls. (A) Oxygen consumption (VO_2_). (B) Cardiac output (CO). (C) Right ventricular ejection fraction (RVEF). (D) Left ventricular ejection fraction (LVEF).

While exercising on the conventional cycle ergometer, healthy controls had significantly higher peak CO (*p* = 0.02) and ΔavO_2_ (*p* = 0.001) than patients. However, there were no differences in peak CO (*p* = 0.15) and ΔavO_2_ (*p* = 0.10) between patients and healthy controls using the novel device (Figure 4B). This was despite both CO and ΔavO_2_ increasing significantly in patient and control groups using both exercise devices (*p* < 0.001).

Peak RVEF was significantly lower, in patients compared with controls using both devices (*p* = 0.02, Figure 4C). However, RVESV only significantly decreased (*p* < 0.01) in healthy controls compared with patients using the novel device. Conversely, RVEDV did not significantly change in patients or controls using the novel apparatus but did fall significantly (*p* = 0.002) in controls using the conventional ergometer.

Peak LVEF was significantly lower in patients compared with controls using the novel exercise device but not with the conventional ergometer (novel *p* = 0.03; conventional *p* = 0.20, Figure 4D). Peak LVEDV and LVESV were not different between patients and controls with either exercise device.

## 4 Discussion

The main findings of this study were (i) exercise protocols using our novel device produced significant changes in CMR/CPET metrics, (ii) there was moderate correlation in exercise CMR/CPET metrics (peak VO_2_, peak HR, peak SV, peak CO, peak ΔavO_2_ and peak ventricular volumes) evaluated using the novel device and conventional MR‐compatible ergometer and (iii) differences in CMR/CPET metrics between healthy volunteers and patients can be detected using both devices.

Exercise CMR techniques have recently gained increased attention with several studies demonstrating the utility of measuring biventricular function during exercise [1, 15]. This is particularly pertinent to diseases like PAH, where exercise CMR has been shown to be a better predictor of mortality than resting CMR [7]. There are currently two commercially available in‐bore MR‐compatible exercise devices that are used in most studies: the MRI cardiac ergometer (Lode BV, Groningen, the Netherlands) and the cardio step module (Ergospect, Innsbruck, Austria) [5, 11, 16, 17]. Unfortunately, commercially available devices can cost more than $40K and are often difficult to use because of their large size and lack of portability. Several lower‐cost, non‐commercial MR‐compatible exercise devices have been described, including supine cycle ergometers, a compact pedal ergometer and stepper devices [8, 9, 10, 18]. These differ substantially in design, and most report only feasibility data in small, healthy cohorts undergoing exercise CMR alone (without integrated cardiopulmonary exercise testing). To our knowledge, none has been validated head‐to‐head against a commercial MR‐compatible ergometer with simultaneous CPET, which we believe is a strength of our study. Their principal limitations relative to our device include greater construction complexity (frequently requiring custom machining or integrated power‐measuring electronics) and limited portability. Thus, we believe that the main advantage of our device over other non‐commercial systems is that it can be very easily manufactured at very low cost.

We have taken a slightly different approach that aims to significantly simplify both the device and the exercise protocol, which keeps costs low, reduces set‐up time and aids dissemination to other sites. Our low‐cost (~$50), highly portable solution can easily be manufactured with components available in most hardware stores. We have also included photographs and the ramped bleep audio files to allow our device and protocol to be simply replicated. The ease of set‐up (less than a minute) and relatively short protocol means that our approach could easily be added to the end of a routine CMR scan, adding around 10 min.

A vital requirement of introducing a novel MR‐compatible exercise device is comprehensive comparison with a current reference standard commercial MR‐compatible ergometer. One might expect resting CMR metrics to be similar irrespective of the exercise devices being used. This was true for LV and RV volumes, but SV was lower with our novel device. We believe this is due to the legs already being raised for the conventional ergometer protocol, resulting in increased SV [19]. Furthermore, there is well‐recognised variability in the leg raise SV response, and this might explain the only moderate correlation in CO, LVEF and RVEF.

We demonstrated moderate‐to‐good correlations for most peak CMR and CPET parameters between the conventional and novel exercise devices. However, peak VO_2_, SV, CO and ΔavO_2_ were significantly lower with our device, indicating that the two exercise protocols are not interchangeable.

Although this is an important limitation of our device, there are well‐recognised differences between conventional treadmill and cycle CPET, with peak values that are not directly interchangeable [20, 21]. The lower peak values with our novel device were most likely due to two reasons: (i) the peak work on our novel device was only associated with the frequency of motion rather than the resistance and this potentially created a ceiling effect, and (ii) fewer muscles were used while exercising on the novel device, which is also the reason for the similar differences seen between treadmill and cycle testing [22].

However, the fact that exercise metrics correlated is unsurprising as even a simple device will evoke a ‘stress’ response. A more central consideration is whether our novel device can differentiate patients from controls, rather than to simply reproduce the values obtained with a commercial ergometer. It is for this reason that we assessed exercise metrics (elicited on both devices) in healthy controls and patients with PAH.

We demonstrated that our novel device was able to elicit the differences in CMR/CPET metrics between healthy controls and patients with PH, in whom exercise intolerance and RV dysfunction is well documented [5, 23, 24]. We were able to show that both the conventional and novel devices were able to elicit significant differences in peak VO_2_, HR and RVEF between PAH patients and healthy controls. Furthermore, using our novel device we were able to demonstrate a difference in peak LVEF between PAH patients and healthy controls, which was not elicited with the conventional bike device. This is an interesting finding as many of our patients also had systemic sclerosis, and we believe our novel exercise protocol was able to unmask underlying LV disease that has been shown in systemic sclerosis patients [25]. The reason that the conventional bike does not unmask LV dysfunction is not obvious but could result from differences in the mechanics of the exercise performed (legs already raised for conventional exercise) that might affect venous return or afterload. Conversely, we also showed that our novel device was not able to show significant differences between patients and healthy controls in CO and ΔavO_2_. These findings suggest that our novel device would be less suited to studies that aim to elicit differences in tissue oxygen extraction. Nonetheless, we do believe that our cheap and easy to use device can provide physiological ‘stress’ that is sufficient to demonstrate differences in contractile reserve. However, further validation is required to confirm the standardisation of workload and physiological responses across different body habitus and study populations.

## 5 Limitations

The main limitation of this study is that the workload on our device was determined solely by the frequency of motion, without any change in resistance. This means that there is a significant ceiling to the maximum work achievable on our novel device (because the maximum leg frequency is limited in most subjects). Conventional ergometers increase work though increasing resistance, and this may elicit a more physiological response. Nevertheless, we have shown significant increases in oxygen consumption, EF and CO, demonstrating that our method is an adequate stressor in PAH patients. However, a limitation is the potential difference in physiological responses among our older patient population. Certain limitations related to the wearing of the weighted TED stockings should also be noted, as comfort, fit and tolerance may have varied between participants and could have influenced exercise performance. Pulmonary function test data were not collected for both groups in both supine and sitting positions, which would have allowed for a more comprehensive evaluation of positional changes in lung compliance and functional residual capacity. A further limitation was that we only investigated a single empirically chosen exercise ramp protocol for the novel exercise device. It is possible that a different protocol might elicit different physiological responses that more closely mirror conventional bicycle ergometry. However, to test more than one protocol would have been impractical, requiring more than two studies being performed on each participant. Similarly, formal assessment of reproducibility and work‐rate linearity was beyond the scope of the present study, as such analyses would have required additional exercise scans in each participant. Nevertheless, with this protocol we have shown that our novel device is able to elicit differences between patients and controls in peak exercise values, particularly related to contractile reserve. Future studies should investigate protocols that more comprehensively evaluate specific components of the exercise response, including tissue oxygen extraction, myocardial perfusion and regional wall motion abnormalities. More robust assessment of reproducibility, exercise capacity and physiological responses, including comparison with other low‐cost MR‐compatible exercise devices, is also warranted. However, the present study was intentionally designed as a proof‐of‐concept study to establish the feasibility of our simple exercise device and demonstrate its ability to generate clinically meaningful physiological responses during CMR. Having established this, the next important step is a larger, ideally multicentre, evaluation addressing these additional endpoints and providing more definitive evidence for clinical translation. Importantly, the simplicity and low cost of the device should facilitate its deployment across multiple centres, enabling assessment of reproducibility and generalisability across different scanners, institutions and patient populations. Such a multicentre study would have been difficult to justify without first establishing feasibility and providing the proof‐of‐concept data presented here. Such a study would be vital for seeking regulatory approval, as well as modifications to the design that improved infection control but still keep the device cheap, portable and easy to use. A final significant limitation of our study was that there were significant differences in age and gender between the patient and control populations, meaning that differences cannot be totally attributed to PAH. However, in this type of comparative study of exercise protocols, we are more interested in demonstrating differences between groups, irrespective of the aetiology. We are also unable to definitively extend our findings to other important cardiovascular diseases including LV failure and ischaemic heart disease. The relatively small sample size limits the generalisability of our findings, and larger multicentre studies involving broader disease phenotypes are required to validate these results and assess their reproducibility. Thus, future studies should focus on diversifying patient populations and potential stress applications. It should also be noted that our design relies on the specific configuration of our scanner that allows the base to fit into a space usually occupied by a cushion. This space is not present on all scanners; hence, we have included an alternative fixation method based on Velcro fastenings (Figure S2). This method should be applicable to most scanner systems and is still low cost and simple. However, with respect to complexity, the simplification offered by our device relates primarily to equipment set‐up, operation and implementation within the MR environment. In some respects, the exercise itself may also be better tolerated than supine cycling, as the reduced range of leg movement avoids the knees contacting the scanner bore, although participant comfort and exercise burden may still vary.

## 6 Conclusions

We have created a simple, easy to use and affordable exercise apparatus for the clinical CMR environment. Although not interchangeable with MR‐compatible supine ergometry, peak exercise metrics correlate reasonably well, and our method is able to detect differences between patient and control groups. Given its portability and low cost to build, this may encourage greater dissemination of exercise CMR in clinical and research practice.

## Funding

D.S.K. is supported by a British Heart Foundation (BHF) Clinical Research Leave Fellowship (FS/CRLF/20/23004) and by the National Institute for Health Research (NIHR) University College London Hospitals (UCLH) Biomedical Research Centre (BRC). M.F. is supported by a BHF Intermediate Fellowship (FS/18/21/33447).

## Ethics Statement

The study was approved by UK National Health Service, Health Research Authority, Research Ethics Committee, and the study conformed to the declaration of Helsinki (IRAS project ID 226101; REC reference 17/LO/1499, National Health Service Health Research Authority UK CRN 058274).

## Conflicts of Interest

There are no conflicts of interest in relation to the work in this manuscript. Nonconflicting relationships with industry are detailed as follows: D.S.K. reports advisory board fees from MSD, and speaker and advisory board fees, research funding, and support for attending meetings from Janssen. T.K. reports speaker bureau fees from Janssen and Inari. M.F. reports consulting income from Intellia, Novo Nordisk, Pfizer, Eidos, Prothena, Alnylam, Alexion, Janssen and Ionis. J.G.C. reports consulting fees from Acceleron; consulting and speaker bureau fees from Bayer, GSK and Johnson & Johnson; and research funding from Johnson & Johnson. R.V. reports support for attending meetings and travel from Janssen.

## Supporting information


**Figure S1:** Measurements of the novel exercise apparatus. (A) 83.3, (B) 11.2, (C) 31.5, (D) 38, (E) 48.4, (F) 3.8 and (G) 10.5 cm.
**Figure S2:** Heavy‐duty Velcro attachments for fixating the device to the bed.
**Figure S3:** Ramped protocol.

**Video S1:** Healthy control exercising with the novel exercise apparatus moving their legs back and forth in time with a metronome of gradually increasing speed.

**Audio S1** Eighteen‐minute file with bleeps increasing in frequency.

## Data Availability

The data that support the findings of this study are available on request from the corresponding author. The data are not publicly available due to privacy or ethical restrictions.
